# Sustainable Pretreatment of Lignocellulosic Biomass for Biohydrogen Production

**DOI:** 10.3390/molecules31152579

**Published:** 2026-07-24

**Authors:** Ioannis Panagiotopoulos, Donald Huisingh, Emmanuel Koukios

**Affiliations:** 1 Bioresource Technology Unit, School of Chemical Engineering, National Technical University of Athens, Zografou Campus, GR-15700 Athens, Greece; 2 Institute for a Secure and Sustainable Environment (ISSE), University of Tennessee, 311 Conference Center Building, Knoxville, TN 37996-4134, USA

**Keywords:** biological hydrogen production, thermophilic fermentation, biomass pretreatment, agricultural residues, energy crops, forestry residues, hydrogen yield

## Abstract

Biological hydrogen production from lignocellulosic residues is increasingly recognized as a promising route toward sustainable fuel production. However, efficient conversion of these materials requires appropriate pretreatment strategies to enhance carbohydrate accessibility while preserving the quality of the resulting hydrolysates for fermentation to hydrogen. To date, most pretreatment studies have primarily emphasized maximizing sugar release and biomass fractionation, often overlooking the critical role of hydrolysate quality and hydrogen fermentability. This review evaluates lignocellulosic biomass pretreatment technologies with a specific focus on their impacts on hydrogen fermentability. Among all of the well-studied pretreatments, only a few are good candidates for biohydrogen production from lignocellulosic biomass. In particular, the selection of an optimal pretreatment approach was shown to depend not only on the physicochemical characteristics of the biomass but also upon the metabolic capabilities and substrate utilization patterns of the microorganisms employed. This article highlights the need for integrated optimization of pretreatment and fermentation processes and identifies key challenges and opportunities for advancing lignocellulosic biohydrogen production.

## 1. Introduction

Hydrogen is considered to be a clean energy carrier because its utilization does not generate carbon dioxide emissions. Currently, hydrogen is predominantly produced from fossil-based resources, especially natural gas, which limits its long-term sustainability. In addition, alternative production routes such as water electrolysis are currently constrained by their high energy requirements. On the other hand, the use of renewable resources such as biomass for hydrogen production [1,2] is more sustainable and is expected to be further developed in the future.

Compared with conventional production pathways, biological processes present notable benefits such as reduced energy demand, relatively high conversion efficiencies and the ability to utilize a wide range of organic waste streams as feedstocks [3,4,5]. These features make such processes well aligned with the principles of sustainability and resource recovery. Biological methods of hydrogen production include the use of algae and cyanobacteria, photosynthetic bacteria and fermentative bacteria.

Fermentative hydrogen production is particularly suitable for processing wet biomass streams, as these substrates can be efficiently converted into hydrogen without the need for extensive drying or energy-intensive preprocessing. A wide variety of organic substrates can be utilized by hydrogen-producing microorganisms, ranging from simple sugars such as hexoses, pentoses, and sucrose to more complex polymers including starch, cellulose, and pectin. In addition, these biological systems can be implemented at relatively small scales, since they do not depend upon large industrial infrastructure to remain economically viable. Among the available biological routes, dark fermentation is especially attractive due to its independence from light energy and its ability to process diverse lignocellulosic and organic waste substrates [6]. Furthermore, dark fermentation systems are typically characterized by simple reactor configurations and comparatively low energy requirements, which are mainly associated with mixing and basic operation [3]. For simplicity, hydrogen produced via dark fermentation is hereafter referred to as biohydrogen.

Extreme thermophilic anaerobic bacteria have attracted considerable attention due to their high yield with respect to hydrogen production. Reported hydrogen yields of around 3 mol/mol hexose have been demonstrated in several studies [7,8] and have been discussed in relevant reviews [9,10,11]. These yields correspond to approximately 75% of the maximal theoretical value of 4 mol hydrogen per mol of C6 sugar. Beyond high hydrogen yields, thermophilic bacteria present metabolic advantages over industrial yeast strains commonly used in ethanol production, as they are capable of converting not only hexose and pentose sugars but also oligomeric carbohydrates into hydrogen. On the other hand, the dark fermentation pathway has economic feasibility issues, which can be addressed by utilizing cheap raw materials, such as lignocellulosic residues.

The conversion of lignocellulosic residues into fermentable substrates requires appropriate pretreatment strategies capable of enabling efficient sugar release for subsequent hydrogen production. In this study, selected pretreatment approaches were evaluated based on their combined performance in terms of carbohydrate recovery, biomass fractionation, and hydrolysate fermentability for hydrogen production. The discussion is limited to pretreatment technologies that have reached a significant level of development or demonstrate clear potential for future industrial application.

## 2. Lignocellulosic Biomass Sources for Biohydrogen Production

Lignocellulosic biomass includes a broad range of materials, including agricultural and forestry residues, agro-industrial by-products, and energy crops along with their residual streams, and it represents a potential feedstock for biological hydrogen production [2,12]. Due to their wide availability and low cost, they are regarded as promising sources of fermentable substrates. However, their practical utilization is constrained by challenges associated with collection, transportation, and processing. Among the individual stages of biohydrogen production, pretreatment typically represents the most cost-intensive step. Therefore, the overall feasibility of cellulosic hydrogen production strongly depends on the choice of feedstock pretreatments enabling efficient and economically viable saccharification and subsequent hydrogen fermentation.

Regarding the technical suitability of a lignocellulosic raw material for hydrogen production, the carbohydrate content and hydrogen fermentability of the raw material are the critical factors. In addition, evaluating both the yields and the potential value of coproducts generated during pretreatment is important when selecting appropriate raw materials [13]. The choice of pretreatment method is generally determined by the specific type and the structural composition of the biomass. To date, agricultural residues, agro-industrial by-products, and energy crops have received the greatest attention for dark fermentative hydrogen production compared with other biomass types. Forestry residues have also been investigated [14,15], and they may be studied more in the future in the context of the biorefinery, which allows for higher lignin valorization.

Some representative examples of agricultural residues that have been reported for dark hydrogen fermentation are wheat straw [16,17,18,19], rice straw [20,21,22], barley straw [23,24], corn stover [25,26,27] and corn cob [28,29]. Energy crops that have been studied for dark hydrogen fermentation are *Miscanthus* [7,30], sweet sorghum [31,32,33], sugarcane [34] and reed canary grass [35]. The residues of some energy crops, such as sweet sorghum bagasse [8,36] and sugarcane bagasse [37,38,39], have also attracted significant attention.

Lastly, various agro-industrial residues such as carrot pulp [40], potato steam peels [41], sugar beet molasses [42], sugar beet pulp [43], cottonseed cake [44], groundnut deoiled cake [45], and palm oil mill effluent [46] have been explored as substrates for dark fermentative hydrogen production. This broad range of candidate feedstocks for biohydrogen production implies that the development of feedstock-specific technologies allowing effective and low-cost saccharification and fermentation will be needed. Hence, selected pretreatment types, which are suitable for different feedstocks, are discussed in Section 3.

## 3. Pretreatment of Lignocellulosic Biomass for Sustainable Conversion to Biohydrogen

Pretreatment is a necessary step to enhance the availability of structural polysaccharides for subsequent biochemical conversion. The degree of carbohydrate accessibility depends on the type of raw material. In the present study, lignocellulosic biomass was investigated; therefore, pretreatment was complex, usually requiring heat and chemicals. The production of biohydrogen from lignocellulose generally involves three main stages: pretreatment, enzymatic hydrolysis, and hydrogen fermentation. Among these, pretreatment is particularly critical because it strongly influences the efficiency of the downstream processing steps. From a process perspective, an optimal pretreatment method should be economically viable while enabling effective fractionation and recovery of biomass components in usable forms. At the same time, it should generate a substrate that can be readily hydrolyzed with low enzyme requirements across a wide range of feedstocks. In practice, however, no single pretreatment technology currently fulfils all these criteria simultaneously. Nevertheless, several methods exhibit distinct advantages, which are discussed below.

Pretreatment approaches can be broadly categorized into mechanical (e.g., milling, grinding), thermal (e.g., steam-based methods), chemical (e.g., acid or alkaline treatments), and their combinations. Agricultural residues are optimally pretreated with either steam [47,48,49,50,51] or dilute acid [52,53,54] pretreatment. Ammonia Fiber Explosion (AFEX) can also be used for low-lignin feedstocks, such as agricultural residues [55,56]. Concerning pretreatment of food waste, acid and alkaline pretreatments are the most widely investigated in the literature [57]. Forestry residues are mainly pretreated with organosolv [58,59,60] or steam pretreatment [61,62,63]. Among forestry feedstocks, hardwoods could also be pretreated with dilute acid [64,65,66]. Typically, dilute acids are used for the pretreatment of agricultural residues [23,67,68].

Below we present the main characteristics of some selected pretreatment methods and we discuss the key work that has been reported so far and the potential of each method for biohydrogen production. It should be noted that pretreatments with no particular relevance to biohydrogen production or small commercial development were not discussed in this article.

### 3.1. Steam Pretreatment

Steam pretreatment is considered an economically attractive option due to its small processing times and relatively low demands in terms of added chemicals and external energy. It has been traditionally regarded as the most suitable pretreatment method for hardwoods, such as poplar [69,70,71], agricultural residues, such as corn stover [69,72], wheat straw [73,74] and rice residues [75,76], and energy crops, such as switchgrass [77]. In these cases, more than 60% of the hemicellulose is recovered and near complete hydrolysis of the water-insoluble cellulosic component is achieved, albeit at typical high cellulase loadings [69,73,78]. The incorporation of catalysts, including sulfur dioxide or dilute sulfuric acid, has been shown to further improve hemicellulose recovery and enhance the digestibility of cellulose, thereby increasing overall sugar yields. Steam pretreatment has also been explored for softwood processing, primarily with an emphasis on maximizing sugar yield [79,80,81]. In addition, compared with other methods such as dilute acid pretreatment, it offers greater flexibility in feedstock handling, as it can accommodate materials like chopped straw and wood chips without the need for extensive size reduction [62,73].

High severities of steam pretreatment, usually reflected by temperatures of 200 °C or more, are typically required in order to solubilize the hemicellulose of the material and to achieve high (>75%) conversion of cellulose to glucose after subsequent enzymatic hydrolysis. These high temperatures result in the undesirable phenomenon of lignin condensation [82] which inhibits complete cellulose conversion mainly due to adsorption of the cellulase enzymes [83]. Although additional posttreatment steps have been proposed to mitigate these effects [84,85,86], their implementation can increase overall chemical consumption and process complexity, without necessarily ensuring efficient separation of the main biomass fractions (hemicellulose, cellulose, and lignin).

The severity of steam pretreatment is often described by the severity factor Ro, facilitating the comparison among the experimental results of different works: Ro = t·exp[(T − 100)/14.75], where t is residence time in minutes and T is pretreatment temperature in °C [87]. The Ro factor has been previously used to try to optimize steam pretreatment of softwood [85], hardwood [69] and corn stover [69]. Previous research on steam pretreatment of Douglas-fir [85] and poplar [69] used an Ro of 3.6 and an Ro range of 3.4 to 4.1, respectively. To achieve even lower severity, the Ro range of 2.4 to 3.5 has also been reported in the steam pretreatment of poplar wood chips [86]. Low severities have also been used by Kabel et al. [88] and Panagiotopoulos et al. [54], but these researchers worked on dilute acid pretreatment of agricultural residues, such as wheat straw and barley straw, which are generally more easily degraded than wood.

Steam pretreatment has been widely investigated as a preparatory step for converting lignocellulosic materials into biohydrogen, with particular emphasis on agricultural residues such as corn stalks [89,90] and corn stover [25,91]. These feedstocks have shown promising performance, with reported hydrogen yields in the range of 2.8–3.0 mol H_2_/mol hexose [25]. The effectiveness of this pretreatment approach is largely attributed to its ability to disrupt the lignocellulosic matrix, leading to the solubilization of hemicellulose and enhanced accessibility of cellulose to enzymatic hydrolysis. As a result, hydrolysates rich in both C5 and C6 sugars can be obtained. Therefore, steam pretreatment is considered particularly suitable for biohydrogen production, as hydrogen-producing microorganisms can metabolize both C5 and C6 sugars (Table 1). For instance, the extreme thermophilic anaerobe *Caldicellulosiruptor saccharolyticus* can simultaneously utilize glucose and xylose [7,8] when these sugars are present in ratios that are expected in hydrolysates from lignocellulosic biomass. In contrast, other species, such as *Thermotoga neapolitana*, tend to preferentially consume glucose when both sugars are available [7]. Interestingly, *C. saccharolyticus* has also been reported to favor xylose when the two substrates are present at similar concentrations [92].

### 3.2. Dilute Acid Pretreatment

Acid pretreatment has been one of the most extensively investigated methods for treating lignocellulosic biomass [93]. Concentrated acids have been traditionally used to treat lignocellulosic materials, but they are toxic and corrosive and their use disturbs the economic feasibility of the biofuel production [94]. Although the use of concentrated acids has mainly been studied in the past for ethanol production, the pretreatment of a hydrolysate of rice straw with concentrated sulfuric acid, resulting in low hydrogen yields, has been reported [95]. In dilute acid pretreatment the acid is mixed with the biomass at a concentration of 0.1–2.0%, and the mixture is held at temperatures between 150 and 200 °C for periods ranging from seconds to minutes. Typically, sulfuric acid is commonly used as the acid of choice [65,67,96,97]. The hemicelluloses are consequently hydrolyzed.

Beyond the hydrolysis of the hemicelluloses, dilute acid pretreatment also leads to the formation and release of a range of by-products that can compromise hydrolysate quality and reduce its suitability for hydrogen fermentation [7,54,98,99]. These compounds are generally referred to as fermentation inhibitors and typically consist of low-molecular-weight organic acids (with acetic acid being the most prevalent), furan derivatives such as furfural (originating mainly from pentose degradation) and 5-hydroxymethylfurfural (HMF, formed from hexose breakdown), as well as various phenolic substances including vanillin, syringaldehyde, and catechol, which are produced from partial depolymerization of lignin [100,101,102].

Acetic acid is derived from the acetyl groups in hemicellulose. Typically, acetic acid concentrations in hydrogen-focused lignocellulose hydrolysates are higher compared to furfural, HMF, formic acid and levulinic acid. In particular, acetic acid can be found in concentrations of 1.0–5.0 g/L. For instance, corn stover pretreated in different dilute sulfuric acid severities resulted in hydrolysates with acetic acid concentrations of 1.6–5.0 g/L [103]. Moreover, an acetic acid release of 4.4 g/L has been reported from the hydrolysis of hemicellulose of corn cob [104]. This concentration may cause a 15% inhibition or more in hydrogen fermentation [105]. Obviously, higher than the aforementioned inhibitor concentrations can be found in studies that have applied harsher pretreatment conditions. For example, pretreatment of agave bagasse with variable temperature or duration, and a varying H_2_SO_4_ concentration resulted in widely different sugar yields and high acetic acid concentration (7.8 g/L) [106]. With HCl, the acetic acid concentration was even higher (8.3 g/L). Notably, in that work, the concentration of other inhibitors such as formic acid and that of the produced sugars were also high.

Furan compounds are primarily generated under acidic conditions, particularly at low-pH environments such as those encountered during acid pretreatments, whereas their formation is typically minimal under alkaline conditions. Among the various inhibitor groups identified in lignocellulosic hydrolysates, furans have been the most extensively investigated due to their strong impact on microbial performance. In hydrolysates used for biohydrogen production, furfural is commonly detected at concentrations ranging from approximately 0.5 to 2.0 g/L, while HMF is usually found at lower levels of about 0.1 to 1.0 g/L. For instance, dilute hydrochloric acid pretreatment of sunflower stalks at 170 °C has been reported to produce hydrolysates containing 1.2 g/L furfural and 0.1 g/L HMF [99]. Similarly, low HMF (0.0–1.0 g/L) and furfural (0.2–0.6 g/L) concentrations have been reported with dilute sulfuric acid pretreatment of wheat straw, barley straw, corn stalk and corn cob at 170 °C [104]. In addition, hydrothermal processing of different agricultural residues within the temperature range of 26–175 °C has been shown to produce furan concentrations generally below inhibitory thresholds for hydrogen fermentation [107]. On the other hand, the growth of the hydrogen-producing bacterium *Caldicellulosiruptor saccharolyticus* showed 50% inhibition with 1–2 g/L of HMF or furfural, whereas *Thermotoga neapolitana* showed similar inhibition only at higher concentrations of 2–4 g/L [7]. Furthermore, *Thermoanaerobacterium thermosaccharolyticum* W16 has been reported to experience strong suppression of hydrogen production at approximately 2.0 g/L furfural and 1.5 g/L HMF [108]. Overall, hydrogen production performance is generally more sensitive to furans such as HMF and furfural than to phenolic compounds, which tend to be more prevalent under alkaline conditions (pH > 8).

Phenolic substances are primarily formed through the partial depolymerization of lignin during pretreatment. In general, vanillin and syringaldehyde are typically less inhibitory than HMF and furfural [109]. Nevertheless, variability exists within phenolic compounds, as vanillin is typically reported to be more inhibitory than syringaldehyde [110,111]. Experimental findings by Quéméneur et al. [17] showed that the separate addition of furfural, HMF, and phenolic compounds (each at 1 g/L) negatively affected hydrogen production in mixed cultures utilizing xylose. Among these inhibitors, HMF and furfural had a more significant impact on hydrogen yields than the phenolic compounds.

Various lignocellulosic biomass feedstocks, mainly agricultural residues, have been dilute acid pretreated for biohydrogen production. Dilute sulfuric acid has mainly been used. For example, corn stover has been pretreated with dilute sulfuric acid for biohydrogen production with *Thermoanaerobacterium thermosaccharolyticum* W16 [26]. The same acid is known to effectively pretreat rice straw for biohydrogen production with *Thermotoga neapolitana* [21], and wheat and barley straw with *Caldicellulosiruptor saccharolyticus* [104]. Pretreatment of an empty fruit bunch of oil palm with dilute sulfuric acid has also given good hydrogen production results [96]. Notably, despite the good efficiency of sulfuric acid for cellulose hydrolysis, the need to control the release of hydrogen production inhibitors and the need to neutralize the pretreatment hydrolysate could restrict the large-scale applicability of this pretreatment type. On the other hand, other acids have also been used. For example, hydrochloric acid has been used in dilute acid pretreatment of coprocessed fruit-and-vegetable waste and corn stover for biohydrogen production [112]. Hydrochloric acid was found to be superior to sulfuric acid in terms of amount and quality of hydrolyzates. On the other hand, sulfuric acid (and phosphoric acid) has been reported to perform better than nitric acid and hydrochloric acid, in terms of hydrogen fermentability [23]. This is in agreement with the study of Monlau et al. [99], who reported low hydrogen fermentability of hydrochloric acid-treated sunflower stalks.

It should be noted that hydrogen fermentability is feedstock-dependent. This means that lignocellulosic raw materials with similar composition that have been treated with identical conditions may show differences in their hydrogen fermentability.

### 3.3. Alkaline Pretreatment

Alkaline agents are frequently used in biomass pretreatment because they are relatively inexpensive and effective at improving the release of fermentable sugars. Commonly used alkalis include sodium hydroxide [113,114,115,116] and calcium hydroxide [117,118], while potassium hydroxide and ammonium hydroxide have also been investigated [119]. Among these, sodium hydroxide is more frequently employed than calcium hydroxide, primarily because of its greater solubility and its stronger impact on enhancing biomass digestibility. Treatment with NaOH induces structural modifications in lignocellulosic materials, including fiber swelling, increased accessible surface area, reduced polymerization degree, and lower crystallinity. Moreover, it facilitates the cleavage of bonds between lignin and carbohydrates and it contributes to partial lignin disruption. As a result, NaOH-based pretreatment has been successfully applied to a range of feedstocks, including *Miscanthus* [120], giant reed [121], sugarcane bagasse [122] and sweet sorghum bagasse [8].

*Miscanthus* has been delignified to an extent of 77% with the addition of 12% NaOH (*w*/*w* dry matter) to the biomass during extrusion at 70 °C [120], whereas sweet sorghum bagasse has been delignified to an extent of 46% with 10% NaOH (*w*/*w* dry matter) and milder pretreatment conditions [8]. Maas [123] has reported a 47% delignification of wheat straw with 12% NaOH. The degree of delignification can be higher than 50% and depends mainly on the alkali level. Delignification determined that up to 15% lime is insignificant. In the case when severe experimental conditions are applied, lime pretreatment can result in significant but still low delignification [117].

The performance of alkaline pretreatment is strongly influenced by the lignin content of the biomass, being generally more effective for low-lignin materials such as agricultural residues, while showing reduced efficiency for lignin-rich substrates like woody biomass. An important objective of an effective pretreatment strategy is the simultaneous recovery of lignin and hemicellulosic fractions [124,125]. In this context, lignin should ideally be obtained in a relatively pure form [126] and with limited water consumption, although current processes still may require dilution steps [127]. A notable drawback of alkaline pretreatment is the partial loss of hemicelluloses into the lignin-rich liquid stream (often referred to as dark liquor), which is not readily fermentable. The extent of this loss is influenced by factors such as the type and concentration of the alkali as well as the nature of the biomass. For example, hemicellulose losses of approximately 11–13% have been reported when applying a NaOH load of 12% (*w*/*w* dry matter) [8,123].

A wide range of lignocellulosic feedstocks have been subjected to alkaline pretreatment for biohydrogen production, with particular emphasis on agricultural residues [19,99] and energy crops [120,128]. The hydrogen fermentability of alkaline pretreated *Miscanthus* hydrolysates has been previously investigated [7]. Poor fermentability of lime-pretreated *Miscanthus* was reported, whereas the fermentation of a NaOH pretreatment hydrolysate yielded 3.2–3.4 mol hydrogen per mol of C6 sugar. Such values rank among the highest reported for hydrogen generation from lignocellulosic hydrolysates (Table 2). Comparable results have been previously reported in a study which optimized pretreatment severity based on fermentability assessments and demonstrated efficient hydrogen production from NaOH-pretreated sweet sorghum bagasse [8]. For reference, the theoretical maximum hydrogen yield from glucose fermentation is 4 mol hydrogen per mol of C6 sugar.

The aforementioned results suggest that the application of alkaline pretreatment has great potential for enhanced hydrogen production. One of the explanations of the good fermentability of the NaOH-pretreated biomass is that with mild pretreatment conditions at relatively low temperatures (70–75 °C) the generation of fermentation inhibitors is avoided (Table 1).

### 3.4. Organosolv Pretreatment

Organosolv treatment is well known for its ability to separate lignocellulosic biomass into fractions that are more amenable to hydrolysis, while also allowing efficient lignin recovery. The method typically employs a combination of water, an organic solvent, and, in some cases, a catalyst under elevated temperature and pressure conditions (around 200 °C and 400 psi). Commonly used solvents include methanol, ethanol and acetone. To enhance pretreatment efficiency, small quantities of mineral acids such as sulfuric acid are often introduced as catalysts [126]. However, under high-temperature conditions, the addition of external acid may not always be required, as organic acids naturally released from the biomass can facilitate cleavage of lignin–carbohydrate linkages [129]. The main advantage of the organosolv pretreatment is that it is a very selective pretreatment method, yielding the separate fractions of dry lignin, an aqueous hemicellulose stream and a relatively pure cellulose fraction [130].

Organosolv is the only pretreatment method that is very effective for the pretreatment of high-lignin lignocellulose materials, such as softwoods [58]. However, its application is limited by several drawbacks, including the relatively high cost of solvents and reagents, as well as the occurrence of side reactions. In particular, acid-catalyzed degradation of released monosaccharides can lead to the formation of compounds such as furfural and HMF, which are known to inhibit certain hydrogen-producing microorganisms [7,109].

Process optimization is essential for organosolv pretreatment, requiring a combined evaluation of (1) the effect of different ethanol organosolv pretreatments on the characteristics of softwood and (2) the fermentability of the produced hydrolyzate to hydrogen. In general, alkaline-catalyzed systems tend to achieve lower hemicellulose removal but also result in reduced formation of inhibitory compounds. In contrast, acid-catalyzed conditions typically enhance hemicellulose solubilization, although they may simultaneously promote the generation of fermentation inhibitors. Although organosolv systems have been explored for various feedstocks, they have not yet been widely applied to agricultural residues. This is reasonable because of the relatively low lignin content of most of these materials and of the cellulase-inhibitory effect [131] of the increased free phenolic groups in the substrate [132] due to the delignification.

Typically, agricultural residues have been optimally pretreated with either steam [47,49] or dilute acid [52,53,54] pretreatment, but the enzyme loadings which were subsequently applied were prohibitively high from a commercialization point of view. Steam pretreatment and dilute acid pretreatment are discussed in detail in this article.

Organosolv pretreatment for hydrogen production purposes is currently in the developmental stage. So far, sporadic attempts have been made in the hydrogen-oriented organosolv pretreatment of various feedstocks, such as birch [133] and rice straw [134]. More progress is expected with the consideration of woody biomass, particularly softwoods, as raw material for dark fermentative hydrogen production. In the past, various organosolv processes, such as the Lignol process [135], have not achieved commercialization, mainly due to challenges including high process costs and energy consumption. Currently, this conversion pathway is gaining attention with the growing interest in lignin valorization as a potential industrial precursor for renewable chemicals. In particular, the relatively high purity, reactivity and narrow polydispersity of organosolv lignin suggest that it would have considerable potential as a feedstock for the production of higher value products such as adhesives, antioxidants, polyurethanes and carbon fibers [136,137,138,139,140,141]. Therefore, the organosolv pretreatment needs to be optimized to simultaneously enhance biohydrogen production and to increase lignin suitability for higher value products.

**Table 2:** Literature overview of fermentation studies with various lignocellulosic feedstocks and agro-industrial residues.

Feedstock	Pretreatment	Microorganism	Max. H_2_ Yield	Reference
			(mol/mol C6 Sugar)	
*Miscanthus*	Alkaline *	*C. saccharolyticus*	3.4	[7]
*Miscanthus*	Alkaline *	*T. neapolitana*	3.2	[7]
Sweet sorghum bagasse	Alkaline *	*C. saccharolyticus*	2.6	[8]
Sugarcane bagasse	Dilute acid *	*C. butyricum*	1.7	[34]
Sugarcane bagasse	Steam acid *	*Clostridium* species	1.2	[142]
Paper sludge	n.a. **	*C. saccharolyticus*	2.1	[143]
Wheat straw	n.a. **	*C. saccharolyticus*	3.8	[37]
Rice straw	Alkaline *	*C. butyricum*	0.8	[20]
Rice straw	Dilute acid *	Mixed cultures	1.1	[22]
Corn stover	Dilute acid *	*T. thermosaccharolyticum* W16	2.2	[26]
Carrot pulp	Enzymatic	*C. saccharolyticus*	2.8	[40]
Carrot pulp	Enzymatic	*T. neapolitana*	2.7	[40]
Potato steam peels	Enzymatic	*C. saccharolyticus*	3.4	[41]

* Followed by enzymatic hydrolysis. ** Not applied.

## 4. Future Perspectives

Future biohydrogen systems should primarily rely upon lignocellulosic feedstocks, although these materials typically require relatively complex pretreatment steps to make them suitable for efficient hydrogen fermentation. Among the various pretreatment strategies, steam explosion and dilute acid processing are often viewed as more effective than many alternative approaches. However, their application should consider the potential release of fermentation inhibitors because some hydrogen-producing microorganisms are not very tolerant to these compounds. Therefore, the severity of the applied pretreatment should be adjusted to such levels that would simultaneously allow for high sugar production and low inhibitor release. Future research will focus on monitoring this release and controlling the effect of inhibitors on fermentability. Moreover, given that to date, hydrogen production in batch mode has been extensively studied, future research will focus on fermentations in continuous mode, potentially with hydrogen removal. 

It is unlikely that one pretreatment method will be suitable for all lignocellulosic raw materials. This means that for biomass with high lignin content (and thus high lignin valorization potential), pretreatment methods, such as organosolv, may be advantageous in case that the C5 sugars cannot be fully fermented by the hydrogen-producing microorganism. Given that cost reduction is needed for biohydrogen commercialization and that this is mainly expected to be achieved by improving the economics of biomass pretreatment, future focus will be on hydrogen-oriented pretreatments with enhanced biomass fractionation.

## Figures and Tables

**Table 1:** Key parameters of sustainable pretreatment of lignocellulosic biomass for biohydrogen production.

Pretreatment	C5 Sugar Release	Inhibitor Release	Lignin Valorization
Dilute Acid	High	High	Low
Acid-catalyzed steam explosion	Very high	Moderate	Moderate
Organosolv	Moderate	Very low	High
Alkaline	Moderate	Low	Good

## Data Availability

The original contributions presented in this study are included in the article. Further inquiries can be directed to the corresponding author.
